# Efficacy and safety of 577 nm subthreshold laser treatment in acute central serous chorioretinopathy

**DOI:** 10.1186/s12886-026-04917-3

**Published:** 2026-05-13

**Authors:** Güzide Akçay, Hatice Selen Kanar, Ulviye Kivrak, Isil Uslubas, Aysu Karatay Arsan

**Affiliations:** 1 Department of Ophthalmology, University of Health Sciences, Kartal Lütfi Kırdar City Hospital, Istanbul, Türkiye; 2 Faculty of Medicine, Acibadem Mehmet Ali Aydinlar University, Istanbul, Turkey; 3 Advanced Neurologıcal Scıences, Istanbul University Institute of Graduate Studies in Health Sciences, İstanbul, Turkey; 4 Kocaeli City Hospital, Kocaeli, Turkey; 5 Department of Ophthalmology, Sancaktepe Şehit Prof. Dr. İlhan Varank Education and Research Hospital, Istanbul, Turkey

**Keywords:** Acute central serous chorioretinopathy, Optic coherence tomography, Subthreshold micropulse laser, Yellow wavelength laser

## Abstract

**Purpose:**

To assess the subthreshold micropulse laser (SML) treatment in patients with acute central serous chorioretinopathy (CSC).

**Materials and methods:**

This retrospective study included 46 eyes from 46 patients with acute CSC. Twenty-five patients were treated with SML, while 21 were observed without intervention. Outcomes evaluated included best corrected visual acuity (BCVA), outer nuclear layer thickness (ONLT), subfoveal choroidal thickness (SFCT), and subretinal fluid (SRF) height. SML was applied using a 577 nm yellow wavelength laser.

**Results:**

In the SML group, BCVA improved significantly from 0.33 ± 0.13 LogMAR at baseline to 0.07 ± 0.05 LogMAR at 6 months (*p* < 0.001). The observation group showed improvement from 0.29 ± 0.13 LogMAR to 0.14 ± 0.16 LogMAR (*p* = 0.011). SFCT decreased significantly in the SML group (460.36 ± 67.91 μm at baseline to 407.44 ± 60.18 μm at 6 months, *p* = 0.013), with no significant change in the observation group. The SML group also showed a significant increase in ONLT (*p* < 0.001), and complete SRF resorption was achieved in all patients, compared to 23.8% in the observation group.

**Conclusion:**

SML treatment is a safe and effective option for managing acute CSC.

**Trial registration number:**

2024/010.99/2/21-27.03.2024, retrospective design.

## Introduction

Central serous chorioretinopathy (CSC) is a retinal disorder characterized by serous detachment of the neurosensory retina, leading to potential vision loss, primarily affecting the working middle-aged group [1]. CSC is typically classified into two forms: acute and chronic, based on the duration of clinical manifestations [2].

Patients commonly present with symptoms such as central vision loss, central scotoma, metamorphopsia, micropsia, and reduced contrast sensitivity due to subretinal fluid in the macula in the acute form or due to both subretinal fluid (SRF) and accompanying retinal pigment epithelial atrophy in the chronic form [3]. The generally accepted approach for the acute form is to observe for 3–6 months [2, 4]. In contrast, there is no universally accepted treatment protocol for chronic CSC, though photodynamic therapy (PDT) remains the most widely used treatment modality [5]. However, with advancements in laser technologies, subthreshold laser systems are gaining popularity as an alternative therapeutic approach [6].

Subthreshold lasers, which are often considered to be non-damaging, act by reducing retinal and surrounding tissue damage through adjustments in laser parameters such as wavelength, power, pulse duration, and repetition rate, when compared to conventional laser treatments [7, 8]. For this reason, in recent years it has become frequently used in the treatment of diseases affecting the macula such as diabetic macular edema, CSC, and macular edema due to retinal vein occlusion.

Although treatment is not recommended in acute CSC, studies have shown that visual prognosis may deteriorate even in acute cases when subretinal fluid persists for prolonged periods [4]. For this reason, some treatment approaches are being considered for acute CSC cases. In this study, we aimed to demonstrate the effectiveness of 577 nm subthreshold micropulse laser (SML) treatment in patients with acute CSC and to compare the outcomes with the acute CSC patient group who did not receive treatment.

## Material and method

This retrospective study included 46 eyes of 46 patients with acute CSC. Of these, 25 patients were in the SML treatment group, and 21 patients were in the observation group. Institutional review board approval (Dr Lütfi Kırdar Kartal City Hospital Local Ethical Committee-2024/010.99/2/21) was obtained, and the study was conducted in accordance with the Declaration of Helsinki. Due to retrospective design, informed consent was not obtained from the participants.

The inclusion criteria can be listed as follows: (1) Patients ≥ 18 aged who have been diagnosed with acute CSC in fundus fluorescein angiography (FFA) and optical coherence tomography (OCT); (2) Patients who have SRF less than 3 months; (3) Patients who followed up at least 6 months. The exclusion criteria can be listed as follows: (1) Patients who have previous CSC history or treatment history for CSC; (2) Patients who have other retina-choroidal diseases, uveitis, and glaucoma.

Due to the retrospective nature of the study, patients were not randomized into the SML treatment or observation groups. All patients presenting to our clinic with acute CSC were informed about SML treatment as a therapeutic option. Based on patient preference following informed discussion, participants were divided into two groups: an observation group and an SML treatment group.

No adjunctive treatments, including topical or oral acetazolamide or mineralocorticoid receptor antagonists (e.g., eplerenone), were administered to any patients in either the SML treatment or observation groups throughout the study period.

All participants underwent a comprehensive ophthalmological examination. Best corrected visual acuity (BCVA) was assessed using the Snellen chart and converted to the logarithm of the minimal angle of resolution (logMAR) scale for statistical analysis. Slit-lamp biomicroscopy and fundoscopy were performed for all patients. FFA was done at both the initial visit and the 6-month follow-up. All OCT measurements were obtained using swept-source OCT (SS-OCT; DRI OCT Triton, Topcon, Japan). Central macular thickness (CMT) and outer nuclear layer thickness (ONLT) were automatically quantified using the device’s built-in automated retinal segmentation algorithm. Subfoveal choroidal thickness (SFCT) and subretinal fluid height (SRFH) were manually measured by two independent experienced ophthalmologists (G.A. and H.S.K) who were masked to the treatment group assignment. Manual measurements were performed using the integrated caliper tool on high-resolution horizontal B-scan images centered on the fovea. For all manually measured parameters, the mean values of the two independent measurements were used for statistical analysis.

In this study, the SML procedure was performed using a 577 nm yellow wavelength laser (YWL) (Quantel Medical, Subliminal Laser System, France). The SML application was guided by FFA with Area Centralis Lens (Volk Optical, Mentor, OH, USA) by two experienced ophthalmologists (G.A. and H.S.K.). The laser procedure commenced by determining the threshold burn power, which was defined as the minimum power causing a barely visible burn at one disc diameter beyond the vascular arcade. The threshold burn power was then reduced by 50% in 577 nm YWL, and the laser was applied to the leakage regions identified on FFA, with a 160-µm spot diameter, 200 ms pulse duration, 5% duty cycle (DC) energy, and a 3 × 3 pattern mode. All patients in both groups (SML and observation groups) were followed up monthly. The BCVA and OCT parameters were recorded and compared for each group.

### Statistical analyses

Statistical analysis was performed using SPSS 23.0. Shapiro-Wilk test was used for detecting of normality distribution of data. The variables in accordance with the normal distribution were expressed as mean±standard deviation (SD), and the differences between groups were analyzed by *t*-test. The intra-group differences were analyzed by paired *t*-test. For categorical variables, the data difference between the two groups was analyzed by the Chi-square test. *P* values less than< 0.05 were accepted as statistically significant.

## Results

A total of 25 patients in the SML treatment group and 21 patients in the observation group were included in the study. There were no statistically significant differences in age, or gender distribution between the two groups. A comparison of baseline demographical and clinical findings is shown in Table 1.

**Table 1:** Comparison of baseline characteristics and findings between two groups

	SML Group (*n* = 25)	Observation Group (*n* = 21)	*P* value
Age	44.12±8.15 (*r*:36–60)	45.86±6.30 (*r*:33–58)	0.45
Gender (Male) (n/%)	16 (64%)	14 (66.6%)	0.51
Duration	36.64±15.30	41.19±11.58	0.15
BCVA (LogMAR)	0.33±0.13	0.29±0.13	0.43
SFCT (µm)	460.36±67.91	448.28±56.71	0.35
SRFH (µm)	119.56±36.87	133.38±49.77	0.29
ONLT (µm)	91.77±8.62	93.38±10.70	0.55

Abbreviations: BCVA: best corrected visual acuity; CMT: central macular thickness; r: range

SFCT: subfoveal choroidal thickness; SRFH: subretinal fluid height. Data are presented as mean± standard deviation

In the SML group, the mean initial BCVA was 0.33±0.13 Log MAR and it improved to 0.08±0.06 Log MAR in the 3rd month and 0.07±0.05 Log MAR in the 6th month, respectively (*p*<0.001). The mean BCVA in the observation group was 0.29±0.13 Log MAR at the baseline and it improved to 0.24±0.18 Log MAR at the 3rd month and 0.14±0.16 Log MAR at the 6th -month visit, respectively (*p* = 0.011). Although no significant difference was observed in baseline BCVA between the two groups, the mean BCVA at both the 3rd month and the 6th month was statistically significantly better in the SML group (*p* = 0.001 and *p* = 0.041, respectively).

In the evaluation of SFCT, the mean SFCT was 460.36±67.91 μm at the inital visit and it decreased to 429.25±53.40 μm at 3rd month and 407.44±60.18 μm at the 6th -month visit (*p* = 0.013). There was no statistically significant change in SFCT in the observation group during the follow-up (*p* = 0.072). In addition, the mean SFCT was found thinner in the SML group compared to the observation group at both the 3rd and 6th month visits (*p* = 0.042 and *p* = 0.038, respectively) (Table 2).

**Table 2:** Changes and comparisons of OCT parameters of both groups during the study

	SML Group (*n* = 25)	Observation Group (*n* = 21)	*P* value
Initial CMT	421.76±49.70	446.23±50.38	0.079
CMT 3rd month	322.40±22.55	341.11±30.22	0.080
CMT 6th month	278.19±20.63	301.28±22.10	0.061
Initial SFCT	460.36±67.91	448.28±56.71	0.35
SFCT 3rd month	429.25±53.40	451.33±62.20	0.042
SFCT 6th month	407.44±60.18	433.15±70.02	0.038
Initial ONLT	91.77±8.62	93.38±10.70	0.55
ONLT 3rd month	95.23 ± 10.02	94.63 ± 9.82	0.81
ONLT 6th month	101.80 ± 9.60	97.22 ± 7.88	0.017

Abbreviations: CMT: central macular thickness; ONLT: outer nuclear layer thickness; SFCT: subfoveal choroidal thickness. Data are presented as mean± standard deviation

At the initial visit, the mean ONLT in the SML group was 91.77 ± 8.62 μm, which improved to 95.23 ± 10.02 μm at 3 months and 101.80 ± 9.60 μm at 6 months (*p* < 0.001). In the observation group, the mean ONLT at baseline was 93.38 ± 10.70 μm, increasing to 94.63 ± 9.82 μm at 3 months and 97.22 ± 7.88 μm at 6 months (*p* = 0.024). While there was no significant difference in mean ONLT between the two groups at baseline and at the 3-month follow-up, the mean ONLT was significantly higher in the SML group than in the observation group at the 6-month follow-up (*p* = 0.017).

Both groups showed a statistically significant decrease in mean CMT, with no significant difference observed between the groups (Table 2).

At the final visit, all patients in the SML group achieved complete SRF resorption, whereas 5 patients (23.8%) in the observation group still had residual SRF. All 5 of these patients subsequently received SML treatment after the completion of the 6-month study period, based on their persistent clinical findings and shared decision-making.

No adverse effects related to the SML treatment were on FFA or OCT during the study period.

## Discussion

In this study, we aimed to compare the effectiveness and safety profile of 577 SML treatment and observation methods in patients with acute CSC. According to the results of our study, better BCVA and better ONLT were obtained with SML treatment in patients with acute CSC compared to observation.

The widely accepted approach for acute CSC is observation, particularly given that many cases resolve spontaneously over time [2]. However, long-term SRF persistence can cause vision loss due to atrophy of the outer retinal tissues and retina pigment epithelium (RPE) [4]. Therefore, treatment approaches with a favorable safety profile can provide rapid resorption of SRF and prevent atrophy of neurosensory retinal tissues. Long et al. reported better visual outcomes with 577 nm SML treatment compared to the observation in patients with acute CSC after 6 months of follow-up [9]. In line with their findings, our study also observed a statistically significant improvement in BCVA in both groups. However, the final mean BCVA was significantly better in the SML treatment group compared to the observation group. These results suggest that early intervention with SML, leading to faster SRF resorption, may yield greater visual gains in patients with acute CSC.

Additionally, we evaluated ONLT using SS-OCT in our study. As is known, the outer nuclear layer (ONL) contains the nuclei of photoreceptors and directly provides information about the viability of photoreceptor cells [10]. In patients with CSC, irreversible vision loss may occur due to thinning of the ONL even if the SRF is resorbed [11]. Ozdemir et al. showed that thinning in the ONL began in less than 3 months in patients with CSC, with symptom durations ranging from 30 days to over 150 days, and emphasized that ONL thinning increased with the prolongation of symptom duration [12]. In our study, a faster improvement in ONLT was observed with SML treatment and the final ONLT value was statistically significantly higher in the SML group than in the observation group This result may be attributed to the rapid resorption of SRF and the subsequent correction of pigment epithelial detachment, which enhances the oxygenation of the photoreceptors and outer retinal layers, thus preventing further atrophy.

CSC is generally considered a self-limiting condition, it can recur and become chronic [2, 13]. Recurrence is observed in 30–50% of the cases within 12 months of the first episode. Many studies have been conducted to determine risk factors or biomarkers for recurrence or chronicity [13–15]. One such factor associated with increased risk is thick choroidal thickness [13, 14]. In their study, Guo et al. found no significant change in SFCT at the 3-month follow-up in patients with acute CSC who were treated with SML [16]. In contrast, Long et al. observed a decrease in SFCT at both 3rd and 6th months following SML treatment [9]. Similarly, in our study, we found a statistically significant decrease in SFCT starting from 3rd month. The reduction in SFCT observed with SML treatment in patients with acute CSC is an encouraging finding; however, given the 6-month follow-up, further studies with longer observation periods are needed to determine if this translates into a reduced rate of recurrence or progression to chronic CSC.

Studies investigating the effectiveness of laser therapy or some medical treatments in patients with acute CSC have increased [13, 17, 18]. Seraj and colleagues emphasized in their meta-analysis that PDT treatment is more effective than anti-VEGF [18]. However, it is well-known that both of these treatments have systemic or ocular side effects. Therefore, treatment methods with reliable side effect profiles such as STL treatments may be considered more rational. In our study, we utilized a 577 nm wavelength laser with micropulse technology. It is known that the YWL is not absorbed by xanthophylls in the macula. Therefore, it is safe to use in leakage areas close to the macula. Consistent with this theoretical advantage, no treatment-related adverse effects were observed in our cohort. However, long-term prospective studies are needed to definitively establish the complete safety profile of SML therapy in CSC.

Our study has several limitations. First, due to its retrospective and non-randomized design, which may have introduced selection bias affecting the internal validity of the results. Second, the limited repeated-measures methodology constrained modeling of longitudinal change; moreover, the 6-month follow-up, while informative, may be insufficient to assess long-term outcomes such as recurrence and chronicity. Third, the relatively small sample size limits the generalizability of our findings, and the inability to evaluate parameters related to the extent of foveal involvement constitutes an additional limitation of our study.

In conclusion, in patients with acute CSC, SML treatment led to complete SRF resorption in all cases within our cohort. Compared to the observation group, the SML-treated patients showed significantly better visual acuity, improved ONLT, and a thinner SFCT. Given its safety profile and efficacy in promoting rapid recovery, SML treatment may offer a rational approach to prevent the progression to chronic CSC and facilitate faster recovery in acute cases; nonetheless, definitive evidence for reducing recurrence or progression will require adequately powered randomized trials with longer follow-up.

## Data Availability

The data that support the findings of this study are available from the corresponding author upon reasonable request.

## Abbreviations

SML: Subthreshold micropulse laser
CSC: Central serous chorioretinopathy
BCVA: Best corrected visual acuity
ONLT: Outer nuclear layer thickness
SFCT: Subfoveal choroidal thickness
SRF: Subretinal fluid
LogMAR: Logarithm of the minimum angle of resolution
PDT: Photodynamic therapy
FFA: Fundus fluorescein angiography
OCT: Optical coherence tomography
YWL: Yellow wavelength laser
SD: Standard deviation
